# Artificial intelligence-based surrogate modeling of hemodynamic indicators in abdominal aortic aneurysms using computational fluid dynamics

**DOI:** 10.1371/journal.pone.0348911

**Published:** 2026-10-28

**Authors:** MyeungHye Suh, Up Huh, Chiseung Lee

**Affiliations:** 1 Department of Biomedical Engineering, Graduate School, Pusan National University, Busan, Republic of Korea; 2 Department of Thoracic and Cardiovascular Surgery, Pusan National University School of Medicine, Biomedical Research Institute, Pusan National University Hospital, Busan, Republic of Korea; 3 Department of Biomedical Engineering, School of Medicine, Pusan National University, Busan, Republic of Korea; 4 In Silico Medicine Lab, Biomedical Research Institute, Pusan National University Hospital, Busan, Republic of Korea; Army Medical University, CHINA

## Abstract

Abdominal aortic aneurysm (AAA) is a progressive dilatative disease of the abdominal aorta with high rupture-related mortality, emphasizing the need for early risk assessment. Conventional anatomical indicators, such as maximum diameter, are insufficient to characterize rupture risk, motivating the incorporation of hemodynamic parameters. Image-based CFD enables the estimation of these parameters but remains clinically limited by high computational cost and long processing times. To overcome these limitations, deep learning–based surrogate models such as MultiViewUNet have recently been proposed, though previous studies were constrained by relatively low spatial resolution and limited coverage of hemodynamic parameters. In this study, we propose MH-UNet, a multi-view, high-resolution surrogate modeling framework that extends the original MultiViewUNet to a higher input resolution and additional hemodynamic parameters. MH-UNet converts three-dimensional AAA geometries into two-dimensional images from multiple viewing angles and predicts TAWSS, OSI, RRT, and ECAP using data from 101 patients. Both single-inference and dual-inference strategies were implemented and compared. Single-inference consistently outperformed dual-inference across all parameters; the cascaded dual-inference scheme amplified errors through the nonlinear OSI and RRT formulations, resulting in negative R² values for OSI, RRT, and ECAP. Among the single-inference predictions, TAWSS was predicted most reliably (R² = 0.803), whereas OSI was the most challenging to predict. Agreement between single-inference predictions and CFD-derived results demonstrates the potential of MH-UNet as a rapid surrogate tool for estimating WSS-derived hemodynamic indices in AAA under fixed boundary conditions.

## 1. Introduction

Cardiovascular disease (CVD) is reported to be a leading cause of approximately 30% of deaths from non-communicable diseases worldwide [1,2]. Among these, abdominal aortic aneurysm (AAA) is one of the most fatal conditions, defined as a dilation of the normal abdominal aortic diameter by more than 50% [3,4]. Although AAA is generally asymptomatic, rupture carries a high fatality rate, with approximately 80% of patients dying as a result [5]. According to the European Society for Vascular Surgery (ESVS) guidelines, surgical treatment is recommended when the diameter of an unruptured aneurysm exceeds 5.5 cm in men and 5.0 cm in women [6–10]. However, rupture risk exists even in AAAs smaller than these thresholds, and conversely, the actual rupture incidence in large AAAs is reported to be about 5.3% [11,12]. Therefore, it is necessary to establish more precise criteria to select patients for preventive surgical intervention. Simply relying on anatomical indicators such as maximum diameter makes it difficult to fully explain the growth patterns or rupture risk of AAA. Accordingly, research on more reliable quantitative alternative markers has been continuously conducted [13,14]. In this process, biomechanics has emerged as a promising approach that can provide functional indicators based on blood flow and tissue mechanics [12,15]. In particular, wall shear stress (WSS)-derived parameters such as time-averaged wall shear stress (TAWSS), oscillatory shear index (OSI), relative residence time (RRT), and endothelial cell activation potential (ECAP) have been shown to be closely related to aneurysm growth, location, and rupture risk [16–21]. Combining these markers with anatomical indicators may aid in the prediction of AAA growth and rupture risk before clinical occurrence [22].

Over the past two decades, advancements in imaging technology and computational mechanics have led to the increasing application of computational fluid dynamics (CFD) for analyzing the hemodynamic characteristics of AAA in the field of cardiovascular medicine [23–27]. CFD enables detailed analysis of spatial and temporal blood flow patterns; however, its clinical utility and applicability in large-scale studies remain limited due to the substantial computational burden. Despite significant advancements in computational technology and resources, the high computational cost still hinders the transition of CFD from a research tool to a clinical decision-making tool [28,29]. To address this issue, recent studies have investigated deep learning–based approaches to accelerate computational hemodynamics simulations [30–34]. Furthermore, machine learning (ML) employing deep learning has been utilized as a low-dimensional surrogate for CFD [35], demonstrating the advantage of greatly improving computational speed while maintaining the desired level of accuracy [15,36]. However, as these surrogate models are inherently data-driven, training requires representative 3D geometries, boundary conditions, and CFD results as ground truth.

To apply deep learning (DL) to CFD prediction, an appropriate approach capable of processing the three-dimensional (3D) shapes used as inputs and outputs is required. However, 3D geometries are represented as graph data structures, which do not reside in Euclidean space and thus are not well suited to traditional neural network architectures. Therefore, a practical method to convert 3D shapes into Euclidean representations applicable to conventional convolutional neural network (CNN) architectures is needed. Among several previous studies, Faisal et al. [37] demonstrated that curvature alone can be used to predict TAWSS through a MultiViewUNet model based on CFD simulations of aortic aneurysms. However, their study has several limitations, which this research aims to address.

To this end, we propose MH-UNet, a multi-view, high-resolution surrogate framework developed by extending the existing MultiViewUNet. Specifically, we sought to improve prediction quality by increasing the input resolution to address the relatively low resolution of the previous study. Moreover, the model’s scope was expanded by predicting additional aneurysm-related parameters including ECAP, OSI, and RRT alongside TAWSS [38–41]. Within the MH-UNet framework, each hemodynamic parameter was predicted directly from curvature using a single-inference strategy, and a cascaded dual-inference strategy—predicting TAWSS first and reusing it to derive OSI, RRT, and ECAP—was additionally implemented and compared to examine how prediction errors propagate through these nonlinear formulations. Furthermore, to verify the reliability and generalizability of these deep learning–based predictions, we employed a variety of metrics, including Element-wise Mean Absolute Error (EMAE), Element-wise Mean Squared Error (EMSE), Element-wise Root Mean Squared Error (ERMSE), Normalized Mean Absolute Error (NMAE), Normalized Mean Squared Error (NMSE), Normalized Root Mean Squared Error (NRMSE), Peak Signal-to-Noise Ratio (EPSNR), and Structural Similarity Index Measure (SSIM) [37,42,43]. Accordingly, this study proposes a novel approach that converts three-dimensional AAA geometries into two-dimensional images from multiple directions. Although the number of nodes may vary across 3D structures, the 2D images are all represented at the same size, minimizing registration errors. Furthermore, this approach can be applied not only to abdominal aortic aneurysms but also to other fields involving 3D geometric studies. In this study, 101 patient-specific aortic geometries were reconstructed, and CFD simulations were performed to derive TAWSS, OSI, RRT, and ECAP for each case. Subsequently, the MH-UNet framework was trained to predict these hemodynamic parameters, with model predictions validated against CFD results.

## 2. Materials and methods

The overall methodological workflow of this study is illustrated in Fig 1. Computed tomography angiography (CTA) data were first collected from 101 patients diagnosed with abdominal aortic aneurysms. Patient-specific geometries were then reconstructed, meshed, and subjected to CFD analysis using ANSYS 2024 R1 (ANSYS Inc., Canonsburg, PA, USA) to derive WSS-related hemodynamic parameters specifically TAWSS, OSI, RRT, and ECAP. These data were subsequently used to train deep learning models, with the dataset divided into training and testing subsets. Finally, curvature-based images were utilized as inputs for 2D image-based prediction of hemodynamic indicators, and model performance was rigorously evaluated using multiple quantitative metrics.

**Fig 1:**
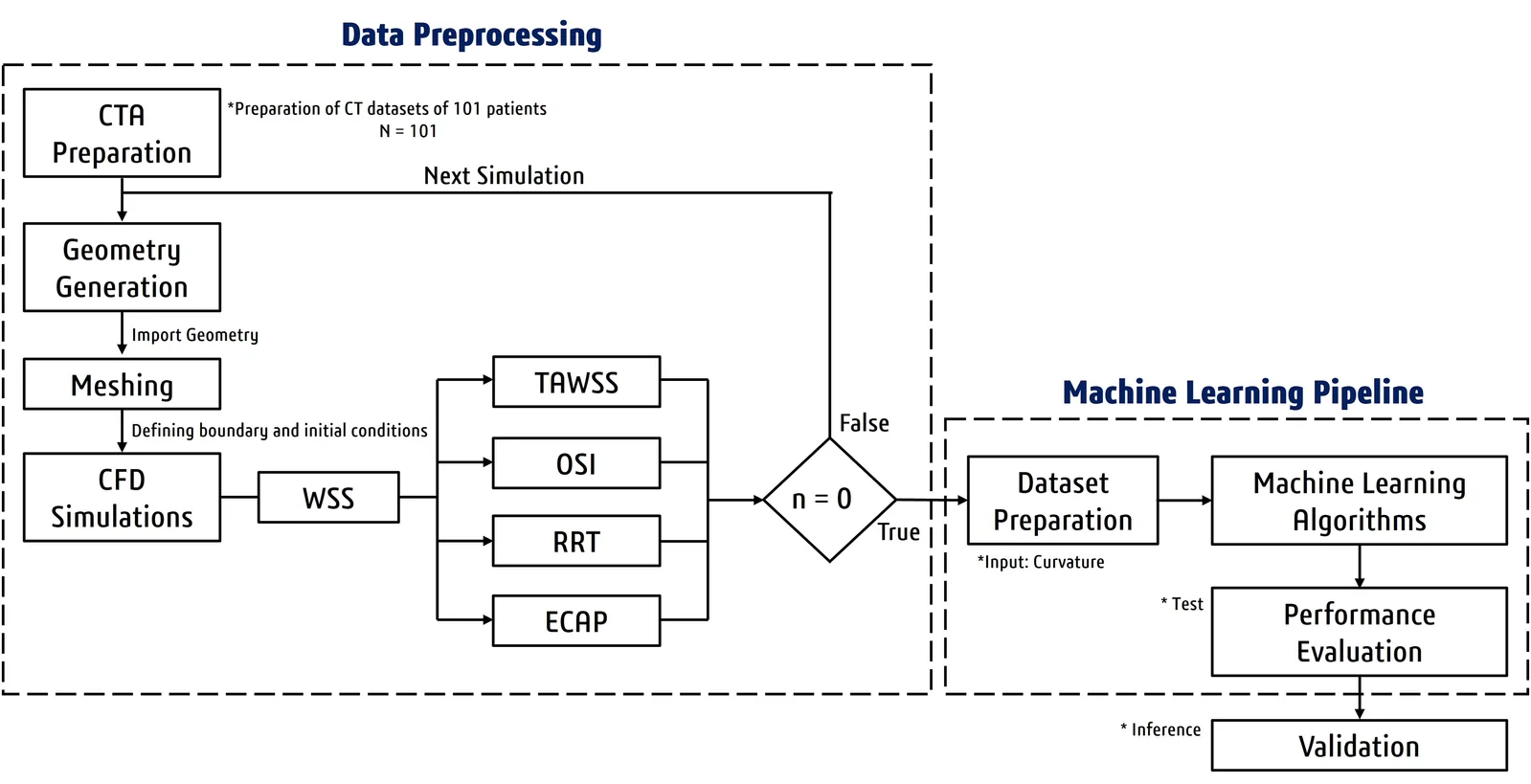
Data processing and machine learning workflow for patient-specific hemodynamic prediction.

### 2.1 Geometry and grid generation

For this study, CTA image data from 101 patients with abdominal aortic aneurysms, acquired at Pusan National University Hospital, were analyzed. The CTA images consisted of 512 × 512pixel matrices, with a slice interval of 2 mm and an average in-plane pixel spacing of 0.785 × 0.785 mm. The raw medical data were transferred to a personal computer and extracted as Digital Imaging and Communications in Medicine (DICOM) files. These files were used for image segmentation and 3D reconstruction using the open-source software 3D Slicer (version 5.6.2). To reduce geometric complexity, arteries other than the infrarenal abdominal aorta and both common iliac arteries were excluded from reconstruction. The resulting vascular surfaces were smoothed using Meshmixer 3.5.474 (Autodesk Inc., San Rafael, CA, USA) to improve morphological fidelity, and subsequently converted into solid models in ANSYS SpaceClaim (ANSYS Inc., Canonsburg, PA, USA) (Fig 2). The medical imaging data used in this study were reviewed and approved by the Institutional Review Board of Pusan National University Hospital (IRB No. 2308-022-130). The data were collected from 23/08/2023–31/10/2023. The requirement for informed consent was waived by the IRB owing to the retrospective design of the study and the use of de-identified imaging data. The computational domain was discretized using ANSYS Fluent Meshing (ANSYS Inc., Canonsburg, PA, USA), generating a tetrahedral grid (Fig 3a). To ensure grid independence, a mesh sensitivity analysis was performed by varying the element count across five levels: approximately 70,000; 170,000; 300,000; 400,000; and 500,000. Key hemodynamic parameters, including average peak systolic velocity, average peak systolic WSS, and maximum peak systolic WSS, were monitored for convergence. The results indicated that convergence was achieved at 400,000 elements; therefore, this grid density was adopted as the optimal standard for all subsequent simulations.

**Fig 2:**
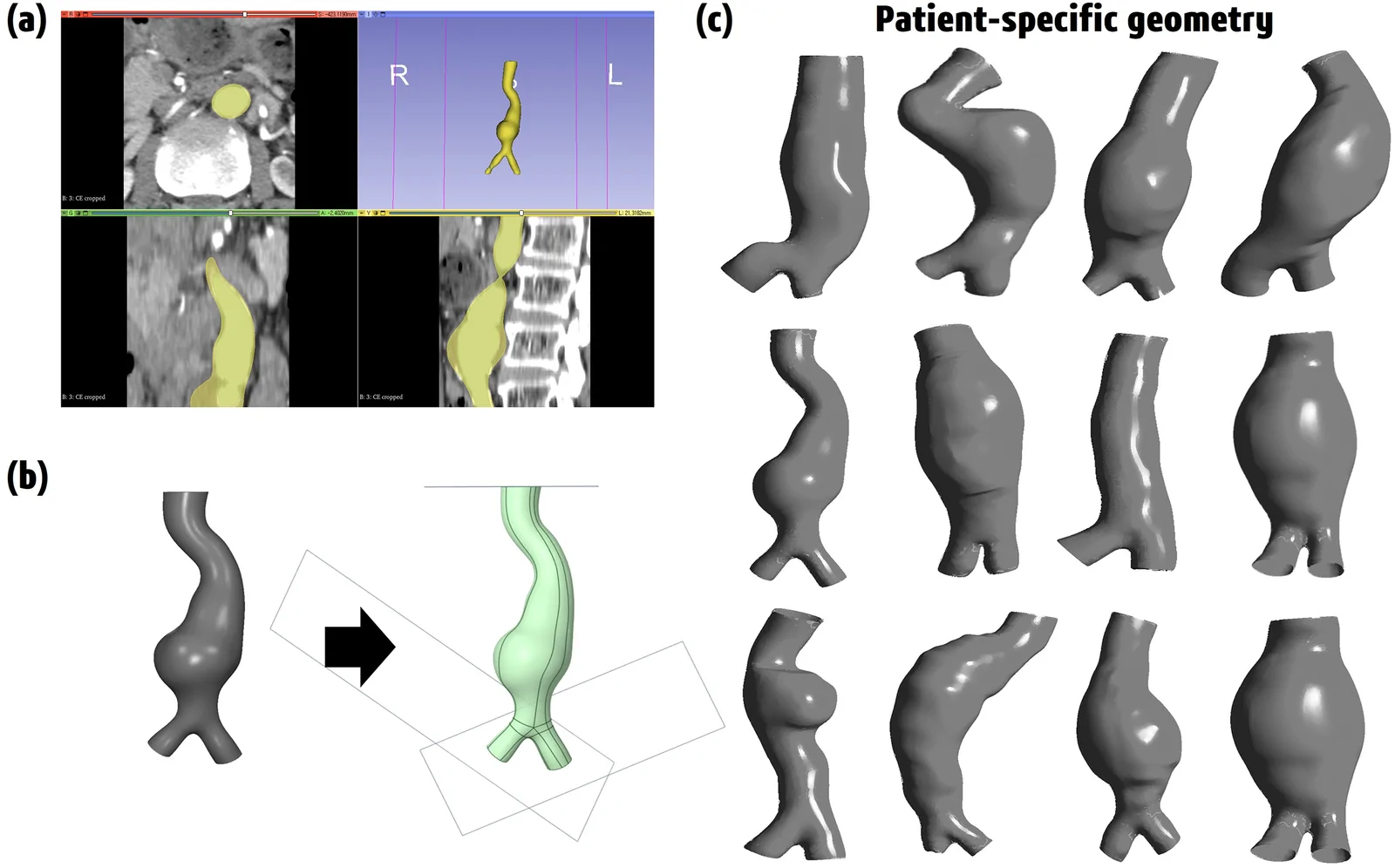
(a) 3D reconstruction of abdominal aortic aneurysm (AAA) geometry from computed tomography (CT) images in Digital Imaging and Communications in Medicine (DICOM) format using 3D Slicer. (b) Surface refinement with Meshmixer followed by conversion to a solid model in ANSYS SpaceClaim. (c) Generation of patient-specific AAA geometries for each case.

**Fig 3:**
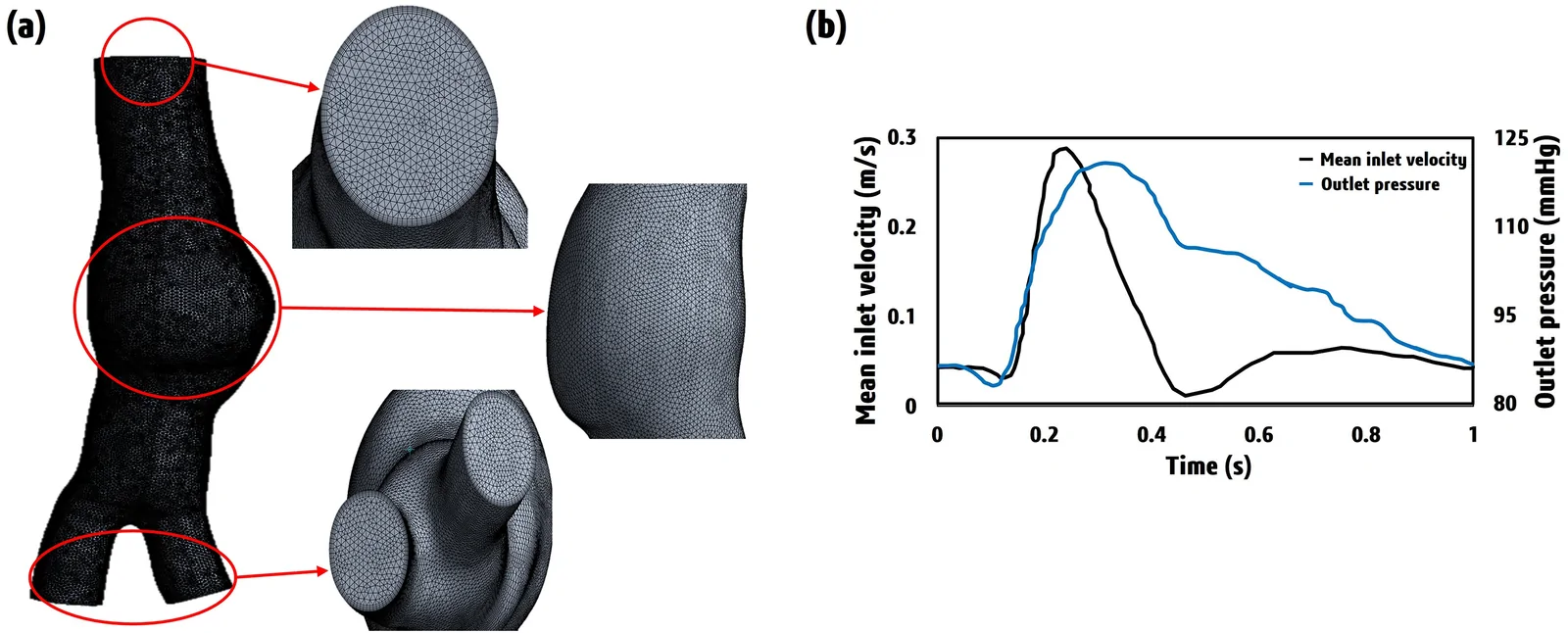
Computational fluid dynamics (CFD) model setup including inlet, outlet, and wall mesh: (a) mesh configuration and (b) inlet velocity profile and outlet pressure profile.

### 2.2 Numerical simulation

For blood flow analysis, the vessel wall was assumed to be rigid, and blood was modeled as a homogeneous, incompressible Newtonian fluid. The fluid density was set to 1050 kg/m³, and the dynamic viscosity was 0.0035 Pa·s [16, 44]. Under these conditions, the governing equations of blood flow are defined by the continuity equation and the Navier–Stokes equations, which are presented in Equations (1) and (2):


$$\begin{array}{c} \frac{\partial u_{i}}{\partial x_{i}}=\text{0}, \end{array}$$ (1)

$$\begin{array}{c} \rho \left(\frac{\partial u_{i}}{\partial t}+u_{j}\frac{\partial u_{i}}{\partial x_{j}}\right)=-\frac{\partial P}{\partial x_{i}}+\mu \frac{\partial^{\text{2}}u_{i}}{{\partial x}_{j}{\partial x}_{j}}+f_{i}, \end{array}$$ (2)

where u represents velocity, P denotes pressure, ρ is the fluid density, and f signifies the body forces acting on the fluid. To analyze the blood flow characteristics in abdominal aortic aneurysms more accurately, the Reynolds number was calculated based on the inlet diameter and velocity. Since the average Reynolds number for each patient was below 2000, laminar flow was assumed. In this hemodynamic environment, parameters derived from WSS—namely TAWSS, OSI, RRT, and ECAP—are key indicators for identifying high-risk rupture regions in AAA. These parameters are defined in Equations (3)–(6).


$$\begin{array}{c} TAWSS=\frac{\text{1}}{T}\left(\int_{\text{0}}^{T}|\tau_{w}|dt\right), \end{array}$$ (3)

$$\begin{array}{c} OSI=\;\frac{\text{1}}{\text{2}}\left(\text{1}-\frac{|\int_{\text{0}}^{T}\tau_{w}dt|}{\int_{\text{0}}^{T}|\tau_{w}|dt}\right), \end{array}$$ (4)

$$\begin{array}{c} RRT=\frac{\text{1}}{(\text{1}-\text{2}\times OSI)\times TAWSS}, \end{array}$$ (5)

$$\begin{array}{c} ECAP=\frac{OSI}{TAWSS}. \end{array}$$ (6)

Among these, TAWSS directly reflects regions of high rupture risk in AAA, where $\tau_{w}$ denotes the wall shear stress vector. OSI characterizes the oscillatory behavior of WSS relative to the predominant axial direction [45]. RRT represents the residence time of blood near the vessel wall, closely associated with flow stasis [46]. Finally, ECAP, derived from both OSI and TAWSS, increases when shear stress exhibits directional inconsistency [47].

### 2.3 Boundary condition settings

The governing equations, Equations (1) and (2), were solved using ANSYS 2024 R1 (ANSYS Inc., Canonsburg, PA, USA). Since blood flow in the aorta is pulsatile, accurate flow modeling requires time-dependent velocity and pressure waveforms. Accordingly, the inlet velocity profile and outlet pressure profile from the cardiac cycle presented by Tzirakis et al. [48] were applied as shown in Fig 3b. The arterial walls were assumed to be stationary with no-slip boundary conditions. The PISO algorithm was used for pressure-velocity coupling [49], and under-relaxation factors of 0.5 were applied for both pressure and momentum equations. The computations were performed on a system equipped with an AMD Ryzen Threadripper 3990X 64-Core Processor (2895 MHz) and 256 GB RAM. To eliminate initial transient effects and obtain stable simulation results, three cardiac cycles were computed, and data from the final cycle were used for analysis. A time step of 0.01s was set, and iterations continued until the residuals for continuity and velocity components in the x-, y-, and z-directions dropped below 10^−4^. From the transient CFD simulations, 300 WSS data points were generated for each condition. For each geometry, only the last cardiac cycle was considered, from which 100 evenly distributed data points were extracted. The temporal average across these 100 time-steps was then calculated to obtain CFD-based distributions of TAWSS, OSI, RRT, and ECAP.

### 2.4 Curvature-based input data for hemodynamic prediction

The AAA geometries used in the CFD simulations were raw geometries. The curvature of these geometries was calculated using PyVista software [50]. Curvature is a fundamental geometric property of a surface that quantifies the rate of change of the tangent vector at a given point. Previous studies have reported a correlation between the curvature at points on the vessel surface and WSS levels. Generally, regions with higher curvature tend to exhibit lower WSS, and it was hypothesized that WSS-related parameters such as TAWSS, OSI, RRT, and ECAP are associated with curvature. Therefore, curvature information was mapped onto each AAA geometry and used as input data.

### 2.5 Computational environment and geometric data generation

The MH-UNet used in this study differs from other machine learning algorithms in that it predicts hemodynamic parameters by using rendered images of geometric structures captured from multiple angles as input. To implement this, a GPU computing environment (NVIDIA GeForce RTX 3060) based on PyCharm was established. Specifically, the environment was configured with Python 3.9 (Python Software Foundation, USA), CUDA 11.2 (NVIDIA Corporation, Santa Clara, CA, USA), TensorFlow 2.10.1 (Google LLC, Mountain View, CA, USA), and cuDNN 11.2 (NVIDIA Corporation, Santa Clara, CA, USA). Independent network training was performed in this environment, demonstrating significantly faster speeds compared to CPU-based computation. Unlike physics-informed neural networks (PINNs), MH-UNet can predict hemodynamic outcomes by transforming 3D geometries into rendered images without relying on physics-based input values. This approach has the advantage of reducing data dimensionality by converting geometric information into images, thereby substantially decreasing computational costs during analysis.

The geometric structures were rendered using PyVista software [50], and through Python scripts, the geometries were rotated around the z-axis in 30-degree increments, capturing snapshot images at each rotation step. The image resolution was maintained at 768 × 768 for use in training. To quantify the node-to-pixel mapping, we compared the number of surface mesh nodes with the number of pixels covering the projected geometry across the dataset. Each geometry contained on average ~21,800 surface nodes (range 15,946–24,213), whereas each rendered view covered ~94,000 pixels at the 768 × 768 resolution (range 57,296–128,536)—approximately four times more pixels than nodes. This indicates that the pixel grid oversamples the surface mesh on average; however, this count-based comparison does not directly quantify the projection error. The process of generating image data from 3D geometries is illustrated in Fig 4.

**Fig 4:**
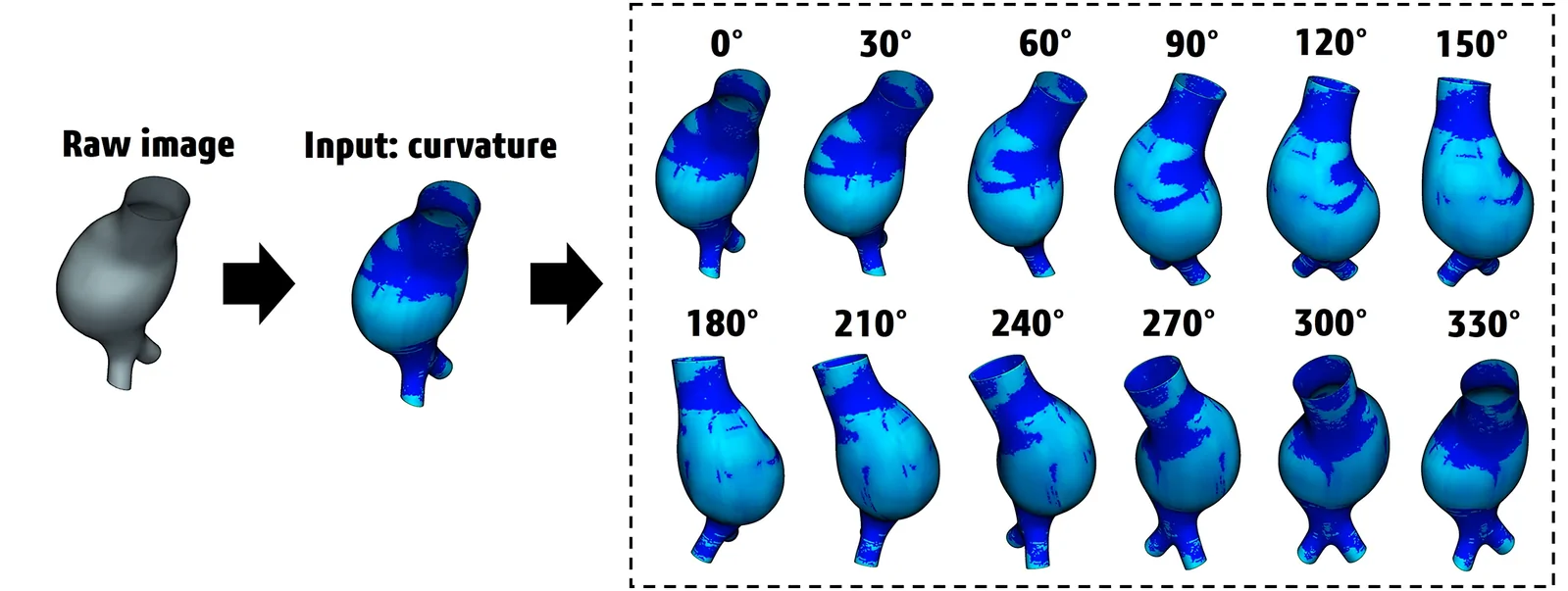
Raw geometry to curvature and generation of images at rotation state around the z-axis.

### 2.6 Image data augmentation

Data augmentation was implemented to expand the training dataset, thereby enhancing the model’s generalization performance and predictive accuracy. During the training phase, identical rotation-based and random zoom augmentations were applied to both the input images and their corresponding ground truth masks to maintain pixel-wise alignment. Specifically, random scaling transformations—ranging from a 10% enlargement to a maximum 70% reduction—were consistently applied using synchronized seed values.

In addition to scaling, spatial augmentations were performed through multi-axis rotations. While the primary rendering was based on the z-axis, additional rotations around the x- and y-axes were introduced to the training set to improve the model’s robustness to various viewing angles. In contrast, the test set was restricted to z-axis rotations to evaluate performance under standardized clinical viewing conditions (Fig 5).

**Fig 5:**
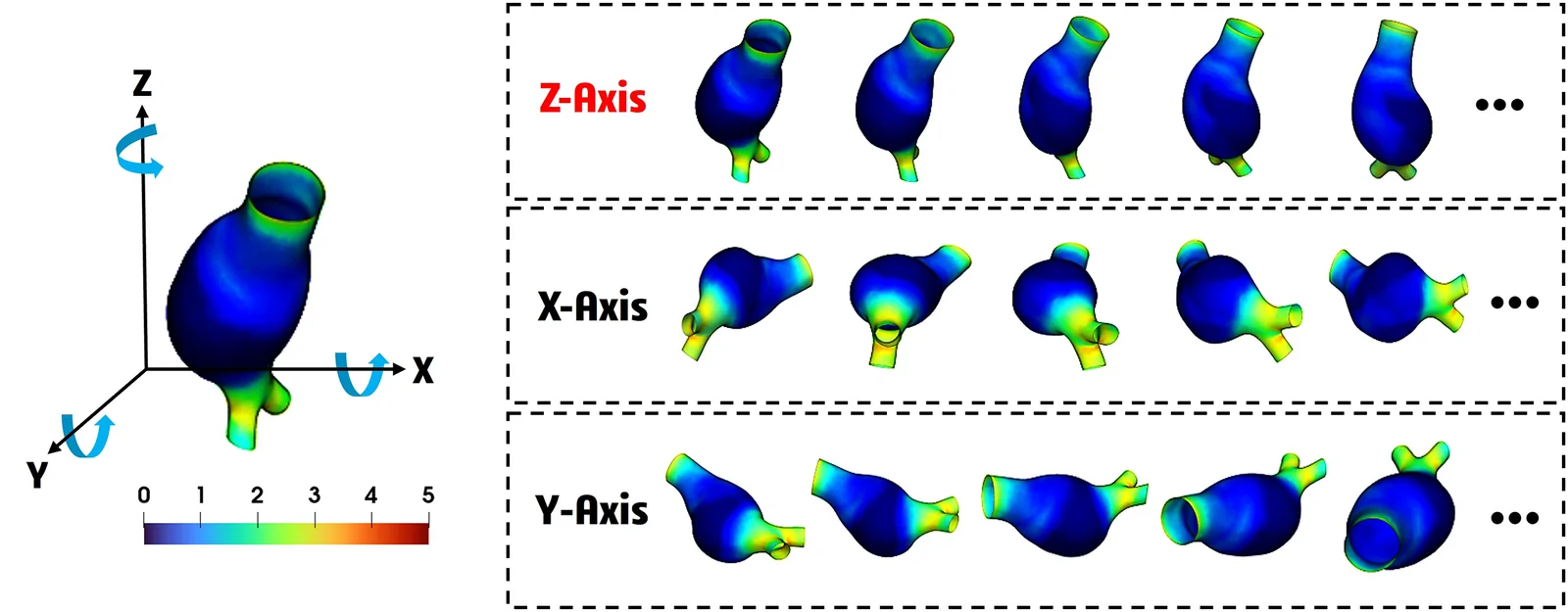
Time-averaged wall shear stress (TAWSS) visualization through incremental 30° rotations of the geometry about the x-, y-, and z-axes.

### 2.7 MH-UNet architecture

The proposed model is named MH-UNet, standing for Multiple Hemodynamic UNet, to emphasize its capability to simultaneously predict various critical hemodynamic indicators (TAWSS, OSI, RRT, and ECAP) essential for AAA risk assessment. The MH-UNet employed in this study extends the conventional UNet based encoder–decoder architecture, enhanced with both inter-skip and intra-skip connections to effectively integrate multi-scale information. The network maps an input image X∈$R^{H\times W\times C}$ to an output Y∈$R^{H\times W\times C}$, and in this study, the input was configured as H = W = 768, C = 3. The high-resolution input (768 x 768) was specifically chosen to capture subtle hemodynamic gradients near the aneurysmal wall, which are often lost in lower-resolution representations. Because a standard U-Net achieved comparable accuracy under the same multi-view, high-resolution pipeline (S1 Table), the inter- and intra-skip connections described below are regarded as an implementation choice rather than the primary contribution of this study.

The network consists of five encoder and decoder blocks, with the number of channels at each stage defined as $n_{d}$=n×${\text{2}}^{d}$. The basic block structure is defined as Equation (7):


$$\begin{array}{c} F_{block}(x)=\;{Conv}_{\text{3}\times \text{3}}\left(BN\left(ReLU\left({Conv}_{\text{3}\times \text{3}}\left(BN\left(ReLU(X)\right)\right)\right)\right)\right), \end{array}$$ (7)

each block adopts a standard convolutional module composed of two 3 × 3 convolution layers, Batch Normalization, and ReLU activation. The global and internal connection structures of the MH-UNet can be categorized into three main types, as shown in Fig 6.

1. Full-scale inter-skip connections: Features extracted from each encoder stage are transmitted to the corresponding decoder stage. Spatial resolutions are matched using bilinear upsampling or max pooling, and the number of channels is reduced with a 1 × 1 convolution. This enables the decoder to simultaneously exploit multi-resolution information, which is expressed in Equation (8):

**Fig 6:**
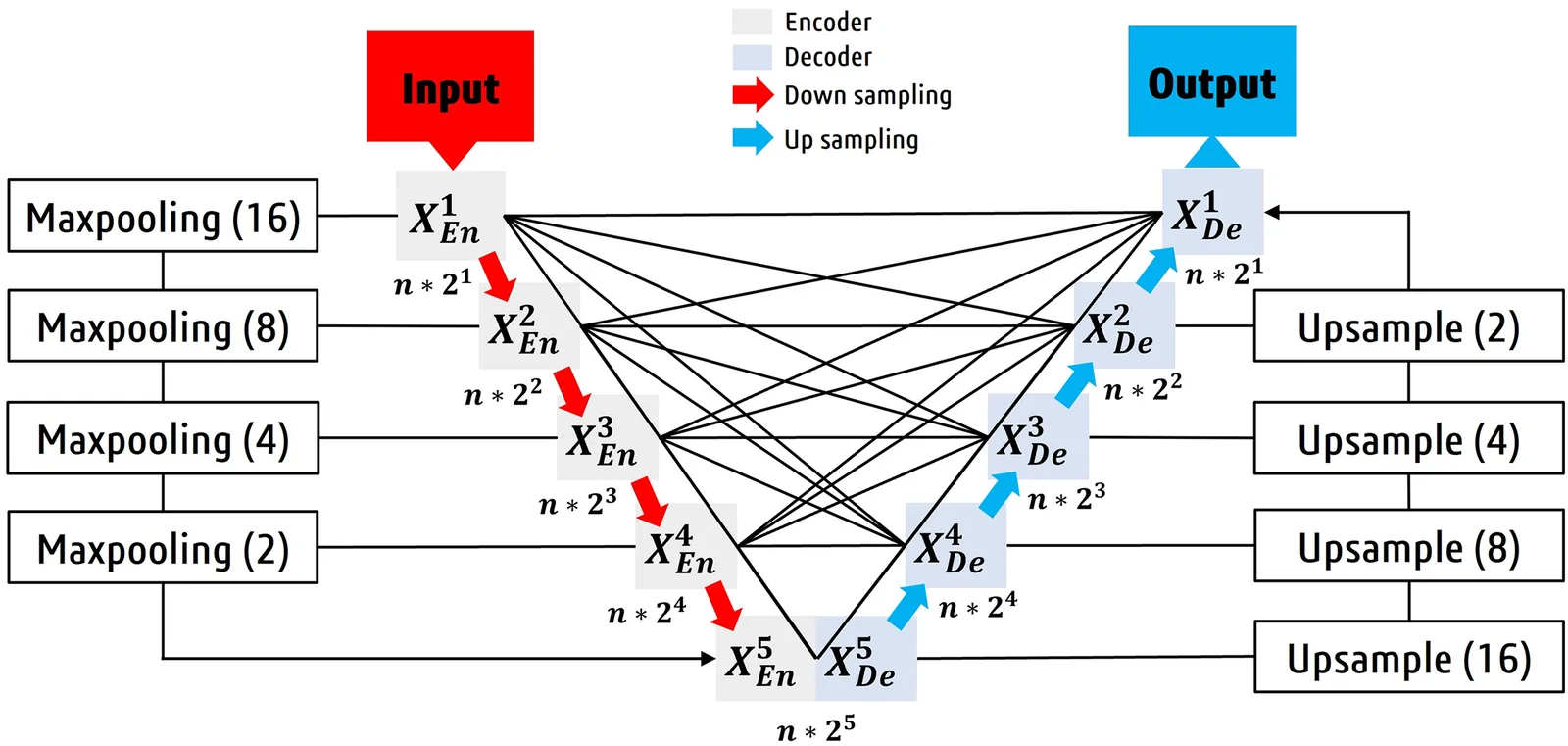
Architecture of MH-UNet, an encoder-decoder network with skip connections.


$$\begin{array}{c} F_{inter}^{d}\;=\;{Conv}_{\text{1}\times \text{1}}(Concat\left[\left\{S{\left(X_{En}^{i},\;d\right\}}_{i=\text{1}}^{d}\right]\right), \end{array}$$ (8)

where the $S(X_{En}^{i},\;d)$ denotes the transformation required to align spatial dimensions of depth d.

2. Encoder intra-skip connections: Within the encoder, outputs from previous stages are cascaded with lower-resolution features to enhance representational capacity at each stage. This process is defined in Equation (9):

$$\begin{array}{c} {\text{X}}_{\text{En}}^{\text{d}}=\text{Conv}(\text{Concat}\left[X_{En}^{d-\text{1}},\left\{\sigma {\left(Maxpool\left(X_{En}^{i},\;{\text{2}}^{d-i}\right)\right\}}_{i=\text{1}}^{d-\text{1}}\right]\right), \end{array}$$ (9)

where the σ represents the sigmoid activation function, and Maxpool(·, k) denotes max pooling with a scale factor of k. This cascaded design enables each stage to integrate multi-scale features and form hierarchical representations.

3. Decoder intra-skip connections: Within the decoder, upsampled features from higher stages are combined to improve fine-grained reconstruction. This operation is expressed in Equation (10):

$$\begin{array}{c} {\text{X}}_{\text{De}}^{\text{d}}=\text{Conv}(\text{Concat}[X_{De}^{d+\text{1}\;},\;\{\sigma (Up(X_{De}^{i},\;{\text{2}}^{i-d}){\}}_{i=d+\text{1}}^{D}]), \end{array}$$ (10)

where Up(·, k) denotes scale-based bilinear upsampling. The upsampled feature maps are passed through a sigmoid-based gating block to reweight their importance, after which multiple feature maps are merged and reduced in channel dimension using a 1 × 1 convolution for efficiency. This hierarchical integration allows the decoder to leverage both spatial details from shallow layers and semantic abstractions from deeper layers, while the gating mechanism facilitates optimal multi-scale feature fusion.

### 2.8 Training and validation

This study utilized data from a total of 101 patients. Among them, data from 53 patients were randomly assigned to the training set, while 24 patients were randomly allocated to the test set and the remaining 24 patients to the validation set for inference. The split was performed at the patient level, so that all rendered views of a given patient belonged to a single subset and no data were shared across subsets.

For the loss function, the NMSE loss was employed. Loss minimization was performed using the Adam optimizer with a learning rate of 0.0001. The maximum number of epochs was set to 300; however, early stopping (patience of 30 epochs on the validation loss) was applied, so that training terminated between epochs 42 and 99 depending on the parameter (Table 2), well before the 300-epoch limit. This, together with batch normalization and data augmentation, was employed to mitigate overfitting given the modest training-set size.

Final model performance was evaluated after training by loading the stored weight file (.weights.h5) for separate inference. Single-inference was conducted using one set of weights, while dual-inference was performed using two sets of weights to derive the results.

### 2.9 Evaluation metrics for prediction results

To quantitatively evaluate the performance of the model, multiple evaluation metrics were employed. The metrics used included EMAE, NMAE, EMSE, NMSE, ERMSE, NRMSE, EPSNR, R², SSIM [37,51]. These metrics collectively assess the model’s prediction error, signal-to-noise quality, and structural similarity. In addition, because RRT exhibits a heavy-tailed distribution near its divergent regions (OSI → 0.5 or TAWSS → 0), the RRT predictions were additionally evaluated on a log-transformed scale, RRT* = log10(1 + RRT). The calculation formulas for each metric are presented in Equations (11)–(19):


$$\begin{array}{c} \text{EMAE}=\frac{\text{1}}{N_{e}}\sum_{i_{e}=\text{1}}^{N_{e}}|Y(i_{e})-\hat{Y}(i_{e})|, \end{array}$$ (11)

$$\begin{array}{c} \text{NMAE}=\frac{EMAE}{max|S|}, \end{array}$$ (12)

$$\begin{array}{c} \text{EMSE}=\frac{\text{1}}{N_{e}}{\sum_{i_{e}=\text{1}}^{N_{e}}(Y(i_{e})-\hat{Y}(i_{e}))}^{\text{2}}, \end{array}$$ (13)

$$\begin{array}{c} \text{NMSE}=\frac{EMSE}{{\left(max|S|\right)}^{\text{2}}}, \end{array}$$ (14)

$$\begin{array}{c} \text{ERMSE}=\sqrt{EMSE}, \end{array}$$ (15)

$$\begin{array}{c} \text{NRMSE}=\frac{ERMSE}{max|S|}, \end{array}$$ (16)

$$\begin{array}{c} \text{EPSNR}=\text{2}\text{0}\cdot {\text{log}}_{\text{1}\text{0}}\left(\frac{{MAX}_{I}}{\sqrt{EMSE}}\right), \end{array}$$ (17)

$$\begin{array}{c} {\text{R}}^{\text{2}}=\text{1}-\frac{\sum_{i_{e}=\text{1}}^{N_{e}}{\left(Y\left(i_{e}\right)-\hat{Y}\left(i_{e}\right)\right)}^{\text{2}}}{\sum_{i_{e}=\text{1}}^{N_{e}}(Y(i_{e})-\overset{―}{Y}{)}^{\text{2}}}, \end{array}$$ (18)

$$\begin{array}{c} \text{ SSIM}(I_{\text{1}},I_{\text{2}})=\frac{\left(\text{2}\mu_{I_{\text{1}}}\mu_{I_{\text{2}}}+a_{\text{1}})(\text{2}\sigma_{I_{\text{1}}I_{\text{2}}}+b_{\text{1}}\right)}{\left(\mu_{I_{\text{1}}}^{\text{2}}+\mu_{I_{\text{2}}}^{\text{2}}+a_{\text{1}})(\sigma_{I_{\text{1}}}^{\text{2}}+\sigma_{I_{\text{2}}}^{\text{2}}+b_{\text{1}}\right)}, \end{array}$$ (19)

where $Y(i_{e})$ denotes the ground truth value at pixel $i_{e},$ and $\hat{Y}\left(i_{e}\right)$ represents the predicted value at the same pixel. $\overset{―}{Y}$ indicates the mean of the ground truth values, while $N_{e}$ refers to the total number of pixels representing the geometric shape. max|S|  denotes the maximum absolute ground-truth value of the corresponding hemodynamic parameter within the vessel region. All three normalized metrics share this normalization basis: NMAE and NRMSE are normalized by max|S|, and NMSE by ${\left(max|S|\right)}^{\text{2}}$; consequently, NRMSE *=* $\sqrt{NMSE}$. ${MAX}_{I}$ denotes the maximum intensity value among all pixels. For SSIM, μ is the mean intensity, σ is the variance, $\sigma_{I_{\text{1}}I_{\text{2}}}$ is the covariance between $I_{\text{1}},I_{\text{2}}$ and $a_{\text{1}}$*,* $b_{\text{1}}$ are small constants introduced for numerical stability.

## 3. Results

The geometric curvature of the AAA was used as input to predict TAWSS, OSI, RRT, and ECAP. As curvature is a key property that reflects the shape and structural characteristics of the geometry, it was used to inform the prediction model. The predictions were compared between two strategies: a single-inference model approach for each hemodynamic index and a sequential dual-inference approach. This comparison was conducted to evaluate the influence of model architecture and inference strategy on prediction accuracy, while the reliability of the results was validated against a reference study (Table 1) [35,42,52–56].

**Table 1:** Summary of representative studies applying deep learning to CFD-based predictions.

REF	Comp. model	Geometry	Learning algorithm	Output	Results
Liang et al., 2020	CFD	729 Thoracic Aortas	DNN	V&P distributions	NMAE: 1.9608%
Rutkowski et al., 2021	CFD	180 Cerebral Aneurysms	CNN	Enhanced 4D flow MRI	RMSE: 2.55- > 1.49, 4.06- > 1.64
Yevtushenko et al., 2021	CFD	228 Coarctation of the Aorta (CoA)	LSTM/ANN	V&P distributions	SSIM Pressure≈6%, WSS ≈ 5%
Wang et al., 2023	CFD	280 Carotid Arteries	Dual-input sampling-channel deep learning network	Pre- and post-procedure predictions for CAS models	NMAE< 7.5%
Zhang et al., 2023	CFD	An aortic model with 88,000 Coronary Artery Branches	PINN	Transient velocity distributions	Velocity field; MAE: 0.015 ~ 0.03 m/s, RMSE: 0.02 ~ 0.05 m/s, Relative Error
Morales et al., 2019	CFD	210 Left Atrial Appendages (LAAs)	MLP	ECAP	NMAE: 4.75%, 5.756%
Li et al., 2021	CFD	Complete Cardiovascular models of 110 patients before and after Coronary Artery Bypass Grafting (CABG)	Point-Net	Pre- and post-procedural velocity distributions	Pre-procedure NMAE: 6.02%, MRE: 9.77% Post-procedure NMAE: 6.01%, MRE: 9.74%

### 3.1 Results based on loss function training

The training results are summarized in Table 2. When predicting TAWSS from curvature, the training loss was 0.0071 and the validation loss was 0.0201, with convergence achieved at epoch 99. For OSI prediction, the single-inference model (curvature → OSI) attained a training loss of 0.0862 and a validation loss of 0.0821, converging at epoch 42. In contrast, the dual-inference model (curvature → TAWSS → OSI) achieved a training loss of 0.0225 and a validation loss of 0.0840, converging at epoch 74. For RRT prediction, the single-inference model (curvature → RRT) produced a training loss of 0.0232 and a validation loss of 0.0532 at epoch 68, whereas the dual-inference model (curvature → TAWSS → RRT) yielded lower losses of 0.0133 (training) and 0.0310 (validation), converging at epoch 81.

**Table 2:** Epoch, training loss, and validation loss of each hemodynamic parameter for single and dual inference modes. Entries marked with a dash (‘–’) indicate that the value was not computed in that mode.

Mode	Variable	Epoch	Training loss	Validation loss
Single-inference	TAWSS	99	0.0071	0.0201
	OSI	42	0.0862	0.0821
	RRT	68	0.0232	0.0532
	ECAP	65	0.0548	0.0842
Dual-inference	TAWSS	–	–	–
	OSI	74	0.0225	0.084
	RRT	81	0.0133	0.031
	ECAP	72	0.04	0.0453

For ECAP prediction, the single-inference model (curvature → ECAP) reached a training loss of 0.0548 and a validation loss of 0.0842 at epoch 65, while the dual-inference model (curvature → TAWSS → ECAP) achieved lower losses of 0.040 (training) and 0.0453 (validation), converging at epoch 72.

### 3.2 Qualitative evaluation of prediction results

Based on the previous training results, inference was performed for TAWSS, OSI, RRT, and ECAP, and the predictions were compared with the ground truth obtained from CFD simulations. The qualitative comparison results are presented in Fig 7. As shown in these figures, although minor discrepancies were observed in some localized regions, the predictions by MH-UNet exhibited an overall distribution pattern that was largely consistent with the CFD simulation results. For TAWSS, a regional breakdown of the prediction error (neck, body, and bifurcation) showed that the error was relatively uniform across regions, with only a modest increase in the aneurysm sac (S1 Fig), where flow is more recirculating and disturbed, indicating that the surrogate does not exhibit a severe localized failure mode.

**Fig 7:**
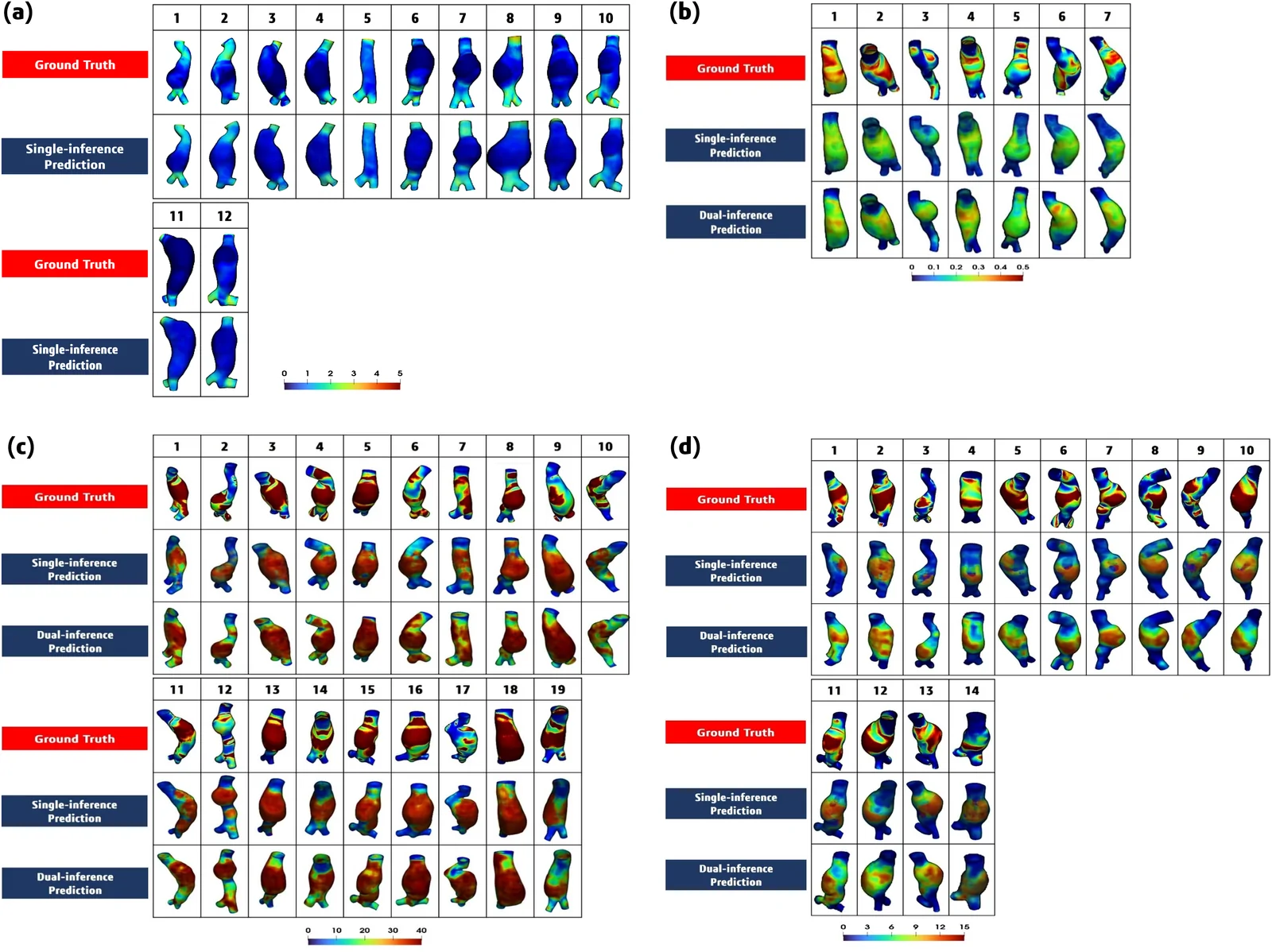
Comparison of hemodynamic parameters obtained from computational fluid dynamics (CFD) simulations (ground truth) with those predicted by the MH-UNet model: (a) time-averaged wall shear stress (TAWSS), (b) oscillatory shear index (OSI), (c) relative residence time (RRT) and (d) endothelial cell activation potential (ECAP). In each panel, the top row shows the CFD ground truth and the lower rows show the single-inference and dual-inference predictions; each column corresponds to one test case. Panel (a) shows single-inference predictions only.

### 3.3 Quantitative evaluation of prediction results

To quantitatively assess prediction performance, various evaluation metrics are summarized in Table 3. Although normalized metrics such as NMAE and NMSE are widely used in this field, they can be distorted for parameters with divergent value ranges such as RRT. Therefore, in this study the element-wise (un-normalized) metrics (EMAE, EMSE, ERMSE) are treated as the primary basis for cross-parameter comparison, with the normalized metrics reported as secondary (Fig 8).

**Table 3:** Quantitative evaluation metrics of hemodynamic parameters for single and dual inference modes.

Mode	Variable	EMAE	EMSE	ERMSE	NMAE	NMSE	NRMSE	EPSNR (dB)	AVG SSIM	R^2^
Single-inference	TAWSS	0.175	0.035	0.187	0.013	0.0004	0.0187	34.82	0.893	0.803
	OSI	0.437	0.138	0.371	0.445	0.289	0.531	5.6	0.807	0.285
	RRT	0.347	0.112	0.335	0.0002	9.0 × 10^−8^	0.0003	89.2	0.783	0.345
	RRT (log)^b^	0.199	0.057	0.239	0.052	0.004	0.062	24.21	0.931	0.276
	ECAP	0.398	0.128	0.358	0.012	0.0002	0.015	36.8	0.787	0.294
Dual-inference	TAWSS^a^	–	–	–	–	–	–	–	–	–
	OSI	0.557	0.288	0.537	0.282	0.148	0.385	8.61	0.956	−0.472
	RRT	0.571	0.287	0.536	0.257	0.146	0.382	8.54	0.952	−0.729
	ECAP	0.527	0.259	0.509	0.265	0.131	0.362	9.07	0.944	−0.389

TAWSS, time-averaged wall shear stress; OSI, oscillatory shear index; RRT, relative residence time; ECAP, endothelial cell activation potential; EMAE, element-wise mean absolute error; EMSE, element-wise mean squared error; ERMSE, element-wise root mean squared error; NMAE, normalized mean absolute error; NMSE, normalized mean squared error; NRMSE, normalized root mean squared error; EPSNR, element-wise peak signal-to-noise ratio; AVG SSIM, average structural similarity index measure over all test-set views; R², coefficient of determination. NMAE and NRMSE are normalized by the maximum absolute ground-truth value, and NMSE by its square.

^a^Dual inference was not applied to TAWSS; –, not applicable.

^b^Metrics computed on log-transformed RRT values.

**Fig 8:**
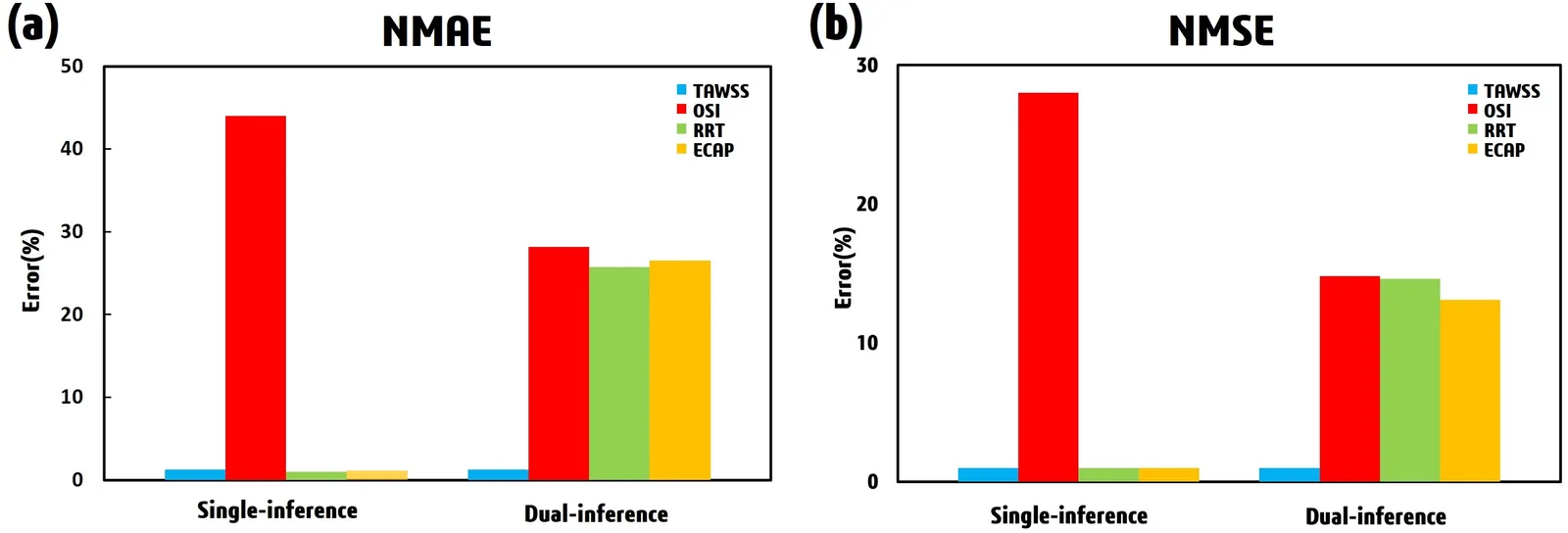
Comparison of error rates between the single- and dual-inference schemes: (a) normalized mean absolute error (NMAE) and (b) normalized mean squared error (NMSE) for the four hemodynamic parameters, time-averaged wall shear stress (TAWSS), oscillatory shear index (OSI), relative residence time (RRT) and endothelial cell activation potential (ECAP).

The dual-inference approach produced larger normalized errors (NMAE, NMSE and NRMSE) than the single-inference approach for RRT and ECAP. For OSI, the normalized errors of the dual-inference approach were lower; however, its element-wise errors were higher (EMAE = 0.557 vs 0.437; ERMSE = 0.537 vs 0.371) and its R² was negative (−0.472), indicating that single-inference remained superior for all parameters. Among the hemodynamic parameters, OSI demonstrated the highest error rate, reflecting lower prediction accuracy, whereas RRT showed the smallest normalized errors. However, this apparently small error of RRT (e.g., NMAE = 0.0002) must be interpreted with caution: RRT diverges as OSI approaches 0.5 or TAWSS approaches zero, so its maximum value—used for normalization—becomes very large and artificially deflates the normalized errors. In un-normalized terms, the element-wise error of RRT (EMAE = 0.347) is comparable to those of ECAP (0.398) and OSI (0.437), indicating no genuine predictive advantage. Consistently, when RRT was re-evaluated on a log-transformed scale, its errors fell in line with the other parameters (NMAE = 0.052, NRMSE = 0.062, EPSNR = 24.21 dB, R² = 0.276). PSNR quantifies the error magnitude relative to the original signal in decibels, whereas EPSNR evaluates how closely the predicted output matches the reference data. Higher EPSNR values indicate that the predictions more closely resemble the original data with better fidelity. In the SSIM analysis, OSI yielded relatively high SSIM values, which can be attributed to its narrow physiological value range (0–0.5). Since SSIM is sensitive to scale and range, small numerical differences are often judged as structurally similar within a restricted data range. By contrast, TAWSS, RRT, and ECAP span broader value ranges, making structural discrepancies more apparent in SSIM.

Finally, R² was used to evaluate the linear correlation between predicted and actual values. SSIM and R² capture different aspects of performance: SSIM measures local structural and luminance similarity within a bounded intensity range and is relatively insensitive to overall magnitude offsets, whereas R² quantifies the fraction of variance explained by point-wise agreement, with negative values indicating performance worse than the training mean. The two metrics can therefore diverge, as observed for dual-inference. In this study, dual-inference for OSI, RRT, and ECAP resulted in negative R² values, indicating that the predictive performance was inferior to simply using the mean of the actual values.

To assess generalization and the possibility of overfitting given the limited training set, the same evaluation metrics were computed on both the training and test sets for all four hemodynamic parameters (Table 4). For every parameter, the test performance was comparable to or better than the training performance (TAWSS R² = 0.803 vs. 0.768; OSI 0.285 vs. 0.158; RRT 0.345 vs. 0.016; ECAP 0.294 vs. 0.154), indicating no overfitting despite the modest training-set size. We note, however, that with only 24 held-out patients these set-level R² estimates carry non-negligible sampling variability; the slightly higher test than training values should therefore be interpreted as evidence for the absence of meaningful overfitting rather than as genuinely superior generalization. The consistently low accuracy of OSI on both the training and test sets further indicates that its weaker performance reflects the inherent difficulty of inferring OSI from curvature rather than overfitting. The larger validation-than-training loss for TAWSS in Table 2 (about threefold) is expected rather than indicative of meaningful overfitting: because the training loss is directly minimized during optimization, it is generally lower than the validation loss, and although the ratio appears large, both values are very small in absolute terms (0.0071 vs. 0.0201). Early stopping (patience = 30) and batch normalization were used to balance model complexity against generalization given the limited sample size.

**Table 4:** Comparison of evaluation metrics on the training and test sets for each hemodynamic parameter (single-inference).

Variable	Set	EMAE	EMSE	ERMSE	NMAE	NMSE	NRMSE	EPSNR (dB)	AVG SSIM	R^2^
TAWSS	Train	0.207	0.049	0.221	0.015	0.0005	0.022	33.36	0.87	0.768
	Test	0.175	0.035	0.187	0.013	0.0004	0.0187	34.82	0.893	0.803
OSI	Train	0.489	0.187	0.432	0.503	0.399	0.623	4.25	0.698	0.158
	Test	0.437	0.138	0.371	0.445	0.289	0.531	5.6	0.807	0.285
RRT	Train	0.474	0.201	0.448	0.0003	1.6 × 10^−7^	0.0004	86.72	0.697	0.016
	Test	0.347	0.112	0.335	0.0002	9.0 × 10^−8^	0.0003	89.2	0.783	0.345
ECAP	Train	0.448	0.175	0.418	0.0132	0.0003	0.017	35.44	0.71	0.154
	Test	0.398	0.128	0.358	0.012	0.0002	0.015	36.8	0.787	0.294

Train, training set: 53 patients, 1,908 views (36 views per patient, rendered at 30° increments about the z-, x- and y-axes).

Test, held-out test set: 24 patients, 288 views (12 views per patient, rendered at 30° increments about the z-axis); not used for model training.

The data were split at the patient level, so no patient appears in both sets.

Hemodynamic parameters: TAWSS, time-averaged wall shear stress; OSI, oscillatory shear index; RRT, relative residence time; ECAP, endothelial cell activation potential.

Error metrics: EMAE, EMSE and ERMSE, element-wise mean absolute, mean squared and root mean squared errors; NMAE, NMSE and NRMSE, the corresponding normalized errors (NMAE and NRMSE divided by the maximum absolute ground-truth value, and NMSE by its square).

Other metrics: EPSNR, element-wise peak signal-to-noise ratio; AVG SSIM, structural similarity index measure averaged over all views in each set; R², coefficient of determination.

Taken together, these metrics capture complementary but sometimes divergent aspects of performance: SSIM reflects local structural similarity within a bounded intensity range and can remain high even when R² is negative (as in dual-inference); normalized errors (NMAE, NMSE) can be distorted for parameters with divergent value ranges such as RRT; and R² together with the element-wise (un-normalized) errors provide the most direct measure of point-wise quantitative agreement. We therefore base cross-parameter comparisons primarily on the element-wise metrics and R², and interpret SSIM and the normalized metrics as complementary indicators.

### 3.4 Prediction accuracy by hemodynamic parameter

Fig 9 presents scatter plots comparing the CFD simulation results with the predictions obtained from the MH-UNet algorithm for TAWSS, OSI, RRT, and ECAP. Panels (a), (b), (d), and (f) display the single-inference results, whereas panels (c), (e), and (g) illustrate the dual-inference results.

**Fig 9:**
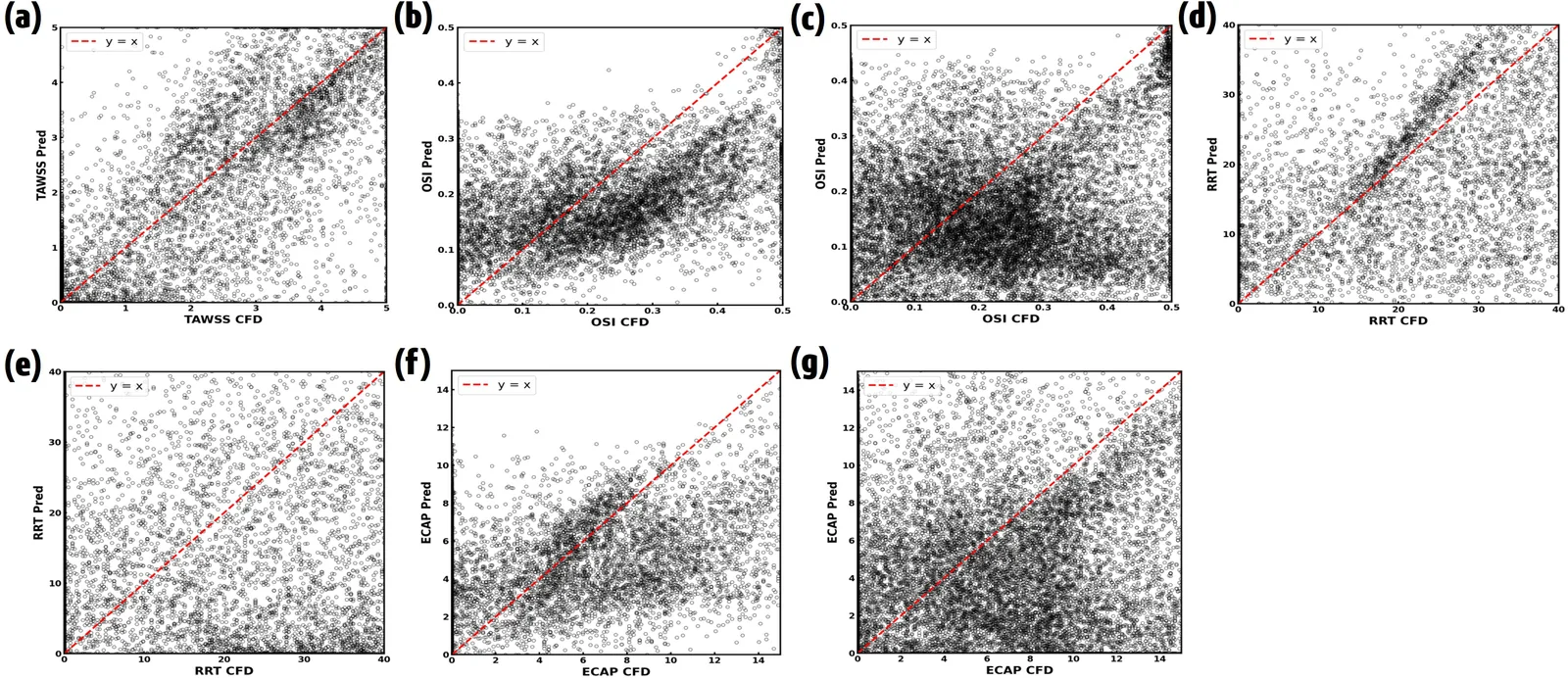
Scatter plots comparing CFD-derived values with MH-UNet predictions. Panels (a), (b), (d) and (f) show single-inference results; panels (c), (e) and (g) show dual-inference results.

Among the evaluated parameters, TAWSS from the single-inference exhibited the highest correlation with the reference CFD values (R² = 0.803), whereas RRT—despite its small normalized errors discussed in Section 3.3—showed only a modest correlation (R² = 0.345). In general, the scatter points from single-inference were more densely distributed along the identity line (y = x), indicating good reproducibility of the predicted values. In contrast, the scatter points from dual-inference were more widely dispersed, reflecting relatively larger prediction errors. These findings suggest that the mapping between input variables and hemodynamic outputs is inherently nonlinear and characterized by complex interactions, which can affect the stability and accuracy of multi-step prediction strategies.

### 3.5 Clinical evaluation of low-WSS region detection

The metrics presented in Sections 3.2–3.4 quantify how closely the predicted fields reproduce the CFD results. However, we considered that these metrics alone may not fully reflect whether the surrogate provides clinically useful information, such as identifying the low-WSS regions relevant to AAA risk. Given the reported association between low WSS and AAA growth and rupture [18], we therefore additionally performed two clinically oriented analyses: (i) whether the surrogate can identify regions below a clinical low-WSS threshold, and (ii) whether its resulting risk-region ranking agrees with CFD. Low-WSS regions were defined as TAWSS < 0.4 Pa, following the established atherogenic threshold [18]. Using the predicted TAWSS as a discriminative score for the CFD-defined low-WSS regions, we constructed a receiver operating characteristic (ROC) curve (Fig 10), which plots the true-positive rate (sensitivity) against the false-positive rate (1 − specificity) as the decision threshold is varied, with the diagonal denoting chance-level discrimination. The surrogate achieved an area under this curve (AUC) of 0.98—where 1.0 indicates perfect and 0.5 random discrimination. Because the AUC corresponds to the probability that a true low-WSS location is ranked as more at-risk than a normal-WSS location, this result also indicates that the surrogate ranks the high-risk (low-shear) regions in strong agreement with CFD; the close spatial correspondence of the predicted and CFD TAWSS fields is likewise evident in Fig 7. At the fixed 0.4 Pa threshold, the surrogate attained a specificity of 0.999, a precision of 0.62, and a Dice coefficient of 0.31 (sensitivity 0.20). The comparatively low sensitivity, together with a moderate case-level correlation of the overall low-WSS burden (Spearman ρ ≈ 0.5), indicates that the absolute extent of low-WSS regions is somewhat under-estimated—an expected consequence of the sparse (~1% of the wall) and conservative threshold rather than a failure of discrimination, given the high AUC. Overall, these analyses suggest that the surrogate localizes and ranks the clinically relevant low-shear (high-risk) regions in reasonable agreement with CFD, while tending to under-estimate their absolute extent.

**Fig 10:**
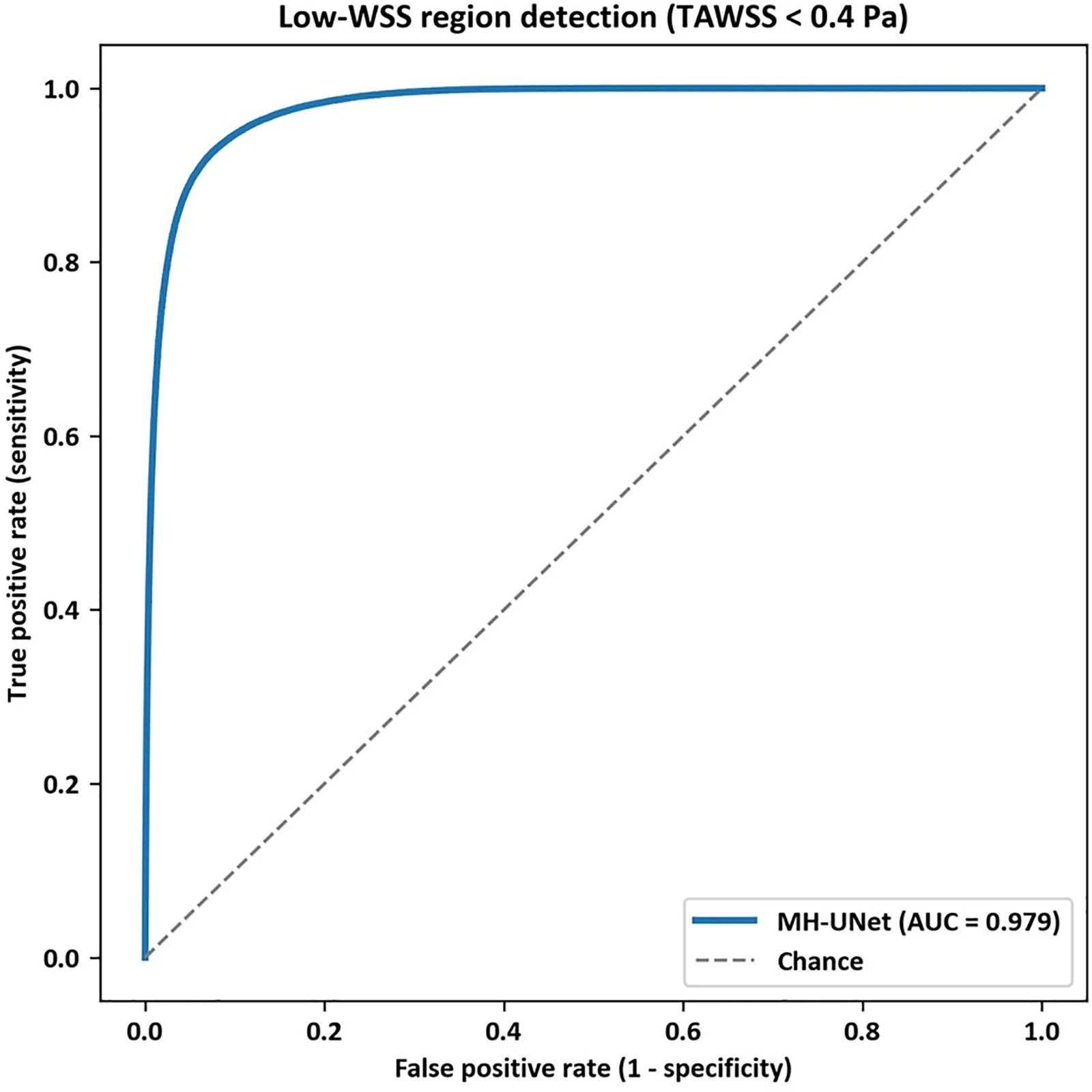
Receiver operating characteristic (ROC) curve for detecting low wall shear stress (WSS) regions defined by computational fluid dynamics (CFD) (time-averaged WSS, TAWSS < 0.4 Pa), using the MH-UNet–predicted TAWSS as the discriminative score over the test set (area under the curve, AUC = 0.98).

## 4. Discussion

In this study, we proposed MH-UNet algorithm as an alternative to computationally intensive and time-consuming CFD simulations using a patient-specific dataset. Recently, AI-based hemodynamic prediction techniques have attracted increasing attention as potential substitutes for CFD in medical imaging and biomechanics. Previous studies have demonstrated the feasibility of deep learning approaches for biomechanical analysis. Liang et al. [57] showed that a deep learning model could accurately predict aortic wall stress distributions within one second as a replacement for finite element analysis. Liu et al. [58] proposed a rapid estimation method for in vivo aortic wall elastic properties. Jaiman [59] utilized CNNs to predict fluid force coefficients across various geometric structures, while Gharleghi et al. [60] developed a CNN-based framework to predict coronary artery WSS, reducing computation time from three hours to two minutes with less than 5% error. Similarly, Su et al. [61] applied CNNs to predict 2D real-time WSS from small clinical datasets. Building upon these prior works, this study employed a modified MultiViewUNet to extend predictions beyond TAWSS to include OSI, RRT, and ECAP. Even when accounting for differences in datasets, the proposed multi-view, high-resolution MH-UNet framework demonstrated substantially lower error rates compared to the benchmarks reported in the original MultiViewUNet study. Specifically, a comparison between the original [37] and MH-UNet for TAWSS prediction showed significant performance improvements, with NMAE decreasing from 0.362 to 0.013, NMSE from 0.021 to 0.0004, NRMSE from 0.643 to 0.0187, and EPSNR increasing from 29.79 dB to 34.82 dB. To examine whether the reported accuracy arises from architectural complexity, we benchmarked MH-UNet against a standard U-Net trained under identical conditions, with the multi-view, high-resolution input pipeline held fixed and only the encoder–decoder backbone changed (S1 Table). The two models achieved comparable accuracy across all four parameters, with MH-UNet yielding marginally higher structural similarity for the more complex parameters (OSI, RRT, and ECAP). This indicates that the predictive accuracy is attributable primarily to the proposed multi-view, high-resolution framework rather than to backbone complexity. The framework is therefore largely backbone-agnostic and can be paired with a lightweight U-Net without loss of accuracy—an advantage for practical deployment. Accordingly, we frame the contribution of this study as the multi-view, high-resolution surrogate framework rather than a specific encoder–decoder design. Because these indices have been associated with AAA growth and rupture risk, extending the prediction beyond TAWSS to OSI, RRT, and ECAP allows these complementary hemodynamic indicators to be estimated rapidly within a single framework. The dual-inference strategy was implemented only as a comparison, not as a proposed method. Single-inference achieved superior accuracy for every parameter, whereas dual-inference produced negative R² values for OSI, RRT, and ECAP (−0.472, −0.729, and −0.389, respectively), indicating performance worse than predicting the training mean. This degradation is an expected consequence of the cascaded design, in which errors in the intermediate TAWSS prediction are amplified when propagated through the nonlinear OSI and RRT formulations. Moreover, this failure is partly inevitable from an information-theoretic standpoint: OSI depends on the directional and phase behavior of the WSS vector, whereas the intermediate TAWSS is a scalar (time-averaged magnitude) from which directional information has already been discarded. Deriving OSI from a scalar TAWSS therefore requires recovering information that no longer exists—an irreversible, non-invertible mapping—so the dual-inference degradation for OSI (and the OSI-derived ECAP and RRT) reflects a fundamental limitation of the cascaded formulation rather than a training deficiency. Although dual-inference yielded high SSIM values (~0.95), this reflects only structural plausibility within a narrow value range rather than quantitative reliability. These results confirm that single-inference is the appropriate configuration for surrogate prediction.

Furthermore, increasing the input resolution from 256 to 768 enhanced the model’s ability to capture subtle hemodynamic patterns, highlighting the importance of resolution in learning complex vascular features.

Despite these promising results, several limitations remain. First, although data from 101 AAA patients were utilized, expanding the dataset could further improve model generalizability. Active learning strategies [62] may also help extract maximal information from limited data. The relatively small training set (53 patients) also introduces a risk of overfitting, as reflected by the gap between the training and validation losses for some parameters (e.g., TAWSS in Table 2). Although early stopping, batch normalization, and augmentation limited this effect—and the held-out test performance remained reasonable (e.g., TAWSS R² = 0.803)—a larger dataset and cross-validation would further improve the balance between model complexity and generalization. Second, the present study assumed a rigid wall for all simulations to ensure computational efficiency. However, actual abdominal aortic aneurysms exhibit elastic properties, and such vessel wall flexibility can significantly influence hemodynamic characteristics. Future research should therefore extend beyond the fixed-wall assumption toward Fluid-Structure Interaction (FSI) models, which simultaneously account for blood flow and vessel wall deformation to achieve more physiologically realistic predictions. Third, identical inlet-velocity and outlet-pressure waveforms were applied to all 101 patients. Because cardiac output, heart rate, blood-pressure waveform, and peripheral resistance vary substantially across AAA patients, this uniform boundary condition introduces a systematic bias: the model effectively learns the geometry–stress relationship for a single fixed flow regime rather than truly patient-specific hemodynamics. Consequently, the surrogate captures geometric (anatomical) patient specificity but not hemodynamic patient specificity, and its predictions should not be interpreted as individualized hemodynamic assessments. Incorporating patient-specific boundary conditions derived from individual physiological signals (e.g., 4D-flow MRI, Doppler ultrasound, or measured blood-pressure waveforms) is therefore an essential next step before clinical application. Fourth, OSI showed the lowest predictive accuracy among the four parameters (NMAE = 0.445, EPSNR = 5.6 dB, R² = 0.285). This likely stems from a fundamental mismatch between the input and the target: curvature is a static, scalar geometric descriptor, whereas OSI is defined by the temporal reversal and directional behavior of the WSS vector over the cardiac cycle. Because the directional and phase information that governs OSI is not uniquely determined by wall curvature, curvature alone is theoretically insufficient to predict OSI, in contrast to the magnitude-dominated and spatially smoother TAWSS, RRT, and ECAP fields; this is further compounded by OSI’s sensitivity to small-scale fluctuations and bidirectional flow in disturbed-flow regions. Consistent with this, previous studies [63] have also reported greater inconsistency and variability in OSI than in TAWSS. We therefore interpret the OSI results with caution and note this as a limitation: reliable OSI prediction likely requires inputs that encode directional and time-resolved flow information (e.g., time-resolved velocity-derived features) rather than geometry alone, and incorporating structural-similarity or adaptive weighting terms in the loss function may further help capture such complex metrics. From the perspective of the learning model itself, these accuracy differences also reflect the nature of the fields and the training objective: TAWSS, RRT, and ECAP are comparatively smooth, magnitude-dominated fields that convolutional networks reproduce structurally, whereas OSI is a high-spatial-frequency field dominated by small, sign-changing fluctuations that convolutional filters tend to smooth out. Moreover, the NMSE-based loss preferentially penalizes large-magnitude errors, favoring the wide-range parameters over the narrow-range OSI (0–0.5), whose compressed range yields a low signal-to-noise ratio for gradient learning. Fifth, the present framework relied solely on image-based data representations without explicitly incorporating physical constraints. Future integration of physics-informed neural networks (PINNs) may enhance both predictive accuracy and reliability by embedding hemodynamic principles into the model. Sixth, while the MH-UNet generated predictions comparable to CFD in most cases, instances of instability or failure were observed in highly complex vascular geometries. This limitation could be addressed through further refinements to the network architecture. Seventh, the parameters predicted in this study—TAWSS, OSI, RRT, and ECAP—are indices closely associated with the growth and rupture risk of AAA. However, at the current stage, this surrogate model provides rapid estimates under fixed boundary conditions and should be regarded as a research tool rather than a clinical risk-assessment instrument. For actual clinical adoption, evaluating generalization performance through multi-center external validation is necessary; this constitutes a crucial step in enhancing the model’s reliability and real-world clinical applicability. Eighth, the 3D-to-2D projection itself may introduce errors that were not quantified in this study. Although the rendered views contained approximately four times more pixels than surface nodes, surface regions that are occluded in all twelve views (e.g., concave regions or the proximal and distal ends) or viewed at oblique angles may be under-represented, and pixel values are interpolated between nodes during rendering. Because all evaluation metrics were computed in the 2D image domain, they do not capture the error incurred when mapping predictions back onto the 3D surface. Future work should quantify this projection error, for example by a round-trip (3D → 2D → 3D) comparison and by measuring surface coverage across views.

## 5. Conclusions

Recent advances in image-based, patient-specific CFD modeling have provided powerful tools for optimizing diagnosis and treatment. Nevertheless, high computational demands and long processing times still limit their use in real-time clinical practice. This study demonstrated that integrating CFD with deep learning can mitigate these limitations by enabling rapid estimation of WSS-derived hemodynamic indices. Such surrogate models may therefore facilitate hemodynamic analysis in large cohorts under the fixed boundary conditions used for training, pending patient-specific boundary conditions and clinical validation. Among the two inference schemes, only single-inference is recommended for practical surrogate use; the cascaded dual-inference scheme was retained solely as a comparison illustrating error amplification in multi-step prediction.

Future work should emphasize large-scale validation across diverse patient populations and disease types to ensure model robustness and generalizability. The proposed approach can extend beyond AAA to other vascular conditions, including thoracic and intracranial aneurysms, providing a versatile framework for rapid hemodynamic estimation.

## Supporting information

S1 Table Comparison between a standard U-Net and MH-UNet (single-inference) under identical training conditions. The two models achieve comparable accuracy across all parameters, confirming the robustness of the proposed framework to the encoder–decoder design.
(DOCX)

S1 Fig Regional distribution of the TAWSS prediction error. Mean absolute error (Pa) in the neck (upper), aneurysm body/sac (middle), and iliac bifurcation (lower) regions, obtained by dividing each geometry into vertical thirds as a simplified surrogate for the anatomical neck, body, and bifurcation regions.
(TIF)
