# Supplementary material for: Artificial intelligence-based surrogate modeling of hemodynamic indicators in abdominal aortic aneurysms using computational fluid dynamics
Source: PLoS One. 2026 Oct 28;21(10):e0348911. doi: 10.1371/journal.pone.0348911 (PMC13716196; doi:10.1371/journal.pone.0348911)
Supplement: Supplementary file 1 [file pone.0348911.s001.docx]

**S1 Table.** **Comparison between a standard U-Net and MH-UNet (single-inference) under identical training conditions.** The two models achieve comparable accuracy across all parameters, confirming the robustness of the proposed framework to the encoder–decoder design.

| **Variable** | **Model** | **EMAE** | **EMSE** | **ERMSE** | **NMAE** | **NMSE** | **NRMSE** | **EPSNR (dB)** | **AVG SSIM** | **R^2^** |
| --- | --- | --- | --- | --- | --- | --- | --- | --- | --- | --- |
| TAWSS | Standard U-Net | 0.172 | 0.036 | 0.190 | 0.0125 | 0.0004 | 0.019 | 34.84 | 0.898 | 0.803 |
|  | MH-UNet | 0.175 | 0.035 | 0.187 | 0.013 | 0.0004 | 0.0187 | 34.82 | 0.893 | 0.803 |
| OSI | Standard U-Net | 0.431 | 0.135 | 0.367 | 0.437 | 0.279 | 0.522 | 5.76 | 0.8 | 0.323 |
|  | MH-UNet | 0.437 | 0.138 | 0.371 | 0.445 | 0.289 | 0.531 | 5.6 | 0.807 | 0.285 |
| RRT | Standard U-Net | 0.341 | 0.106 | 0.326 | 0.0002 | 4.0 × 10⁻⁸ | 0.0002 | 89.52 | 0.759 | 0.323 |
|  | MH-UNet | 0.347 | 0.112 | 0.335 | 0.0002 | 9.0 × 10⁻⁸ | 0.0003 | 89.2 | 0.783 | 0.345 |
| ECAP | Standard U-Net | 0.408 | 0.127 | 0.356 | 0.0119 | 0.0002 | 0.015 | 36.82 | 0.747 | 0.322 |
|  | MH-UNet | 0.398 | 0.128 | 0.358 | 0.012 | 0.0002 | 0.015 | 36.8 | 0.787 | 0.294 |

TAWSS, time-averaged wall shear stress; OSI, oscillatory shear index; RRT, relative residence time; ECAP, endothelial cell activation potential; EMAE, EMSE and ERMSE, element-wise mean absolute, mean squared and root mean squared errors; NMAE, NMSE and NRMSE, the corresponding normalized errors (NMAE and NRMSE divided by the maximum absolute ground-truth value, and NMSE by its square); EPSNR, element-wise peak signal-to-noise ratio; AVG SSIM, structural similarity index measure averaged over all test-set views; R², coefficient of determination.
